# Application of extracorporeal membrane oxygenation in the remote transport of a patient with a ruptured sinus of Valsalva aneurysm: a case report

**DOI:** 10.3389/fsurg.2024.1323614

**Published:** 2024-03-19

**Authors:** Xiaozu Liao, Shi Zhong, Weizhao Huang, Binfei Li

**Affiliations:** ^1^ Department of Anesthesiology, Zhongshan City People’s Hospital, Zhongshan, China; ^2^ Department of Cardiac Surgery, Zhongshan City People’s Hospital, Zhongshan, China

**Keywords:** sinus of Valsalva aneurysm, veno-arterial extracorporeal membrane oxygenation, ECMO, case report, cardiopulmonary bypass (CPB)

## Abstract

**Background:**

A ruptured sinus of Valsalva aneurysm can lead to rapid heart failure and sudden cardiac death. Management of patients who develop severe heart failure and need to be transferred to a specialized hospital for surgical treatment can be challenging. In patients with severe shock due to a ruptured sinus of Valsalva aneurysm into the right atrium, extracorporeal membrane oxygenation (ECMO) transport is an effective means to ensure patient safety, but increases the right cardiac load. We report the experience of veno-arterial (VA) ECMO transport in the treatment of acute cardiogenic shock caused by rupture of a congenital sinus of Valsalva aneurysm.

**Case presentation:**

We describe the case of an 18-year-old male who began having acute episodes of chest pain, shortness of breath, palpitations, and dizziness 18 h before presenting to the emergency department. An echocardiogram revealed an acute ruptured sinus of Valsalva aneurysm and a shunt to the right atrium. The patient presented with severe shock. VA-ECMO was administered to ensure safe transport to the cardiac center. The outcome of emergency surgical repair was good. The patient was on ECMO for 8 h. He returned to the general ward after 7 days and was successfully discharged after 40 days. He had good exercise tolerance 2 years after surgery and no evidence of heart failure.

**Conclusion:**

Although ECMO transport can increase right cardiac load, it is an effective and safe method to move patients with severe shock caused by a ruptured sinus of Valsalva aneurysm into the right atrium. Methods to decrease right cardiac load, such as decreasing ECMO flow combined with cardiotonic drugs, should be adopted. Successful treatment involves rapid establishment of cardiopulmonary bypass and urgent repair of the ruptured sinus of Valsalva aneurysm.

## Background

The incidence of ruptured sinus of Valsalva aneurysm is low, accounting for 0.14%–1.50% of cardiac operations (1). Surgical treatment of ruptured sinus of Valsalva aneurysm has the advantages of low surgical risk and good long-term efficacy. Although it is a high-risk group, early diagnosis and optimal surgical methods can prevent worsening of symptoms and subsequent heart failure (2). Ruptured sinus of Valsalva aneurysms require immediate medical attention and can lead to rapid heart failure, cardiac tamponade, hemodynamic damage, and sudden cardiac death (3). Transferring patients who develop severe heart failure to a specialized hospital for surgical treatment can be challenging. Mechanical support in the form of extracorporeal membrane oxygenation (ECMO) may improve the safety of transporting such patients to a facility with access to advanced medical care (4). We report the case of a patient with acute cardiogenic shock due to a ruptured sinus of Valsalva aneurysm who was transported to our hospital by veno-arterial (VA) ECMO and successfully underwent surgical treatment.

## Case presentation

An 18-year-old male presented to a local hospital with sudden chest pain accompanied by headache, palpitations, and shortness of breath for 18 h. He was in good health, had no previous underlying diseases, and no history of cardiopulmonary disease.

**Figure 1:**
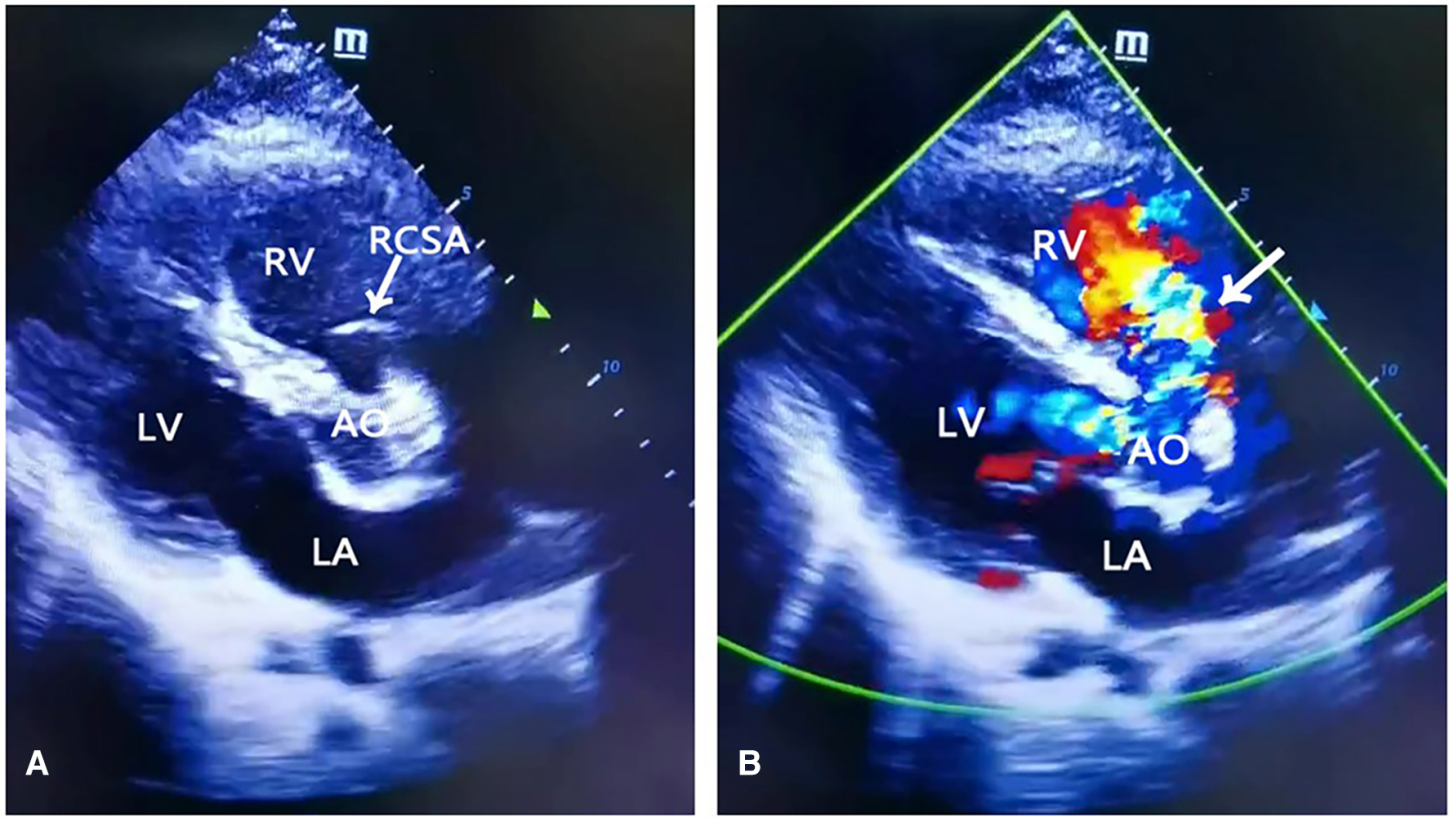
Transthoracic echocardiography. (**A**) Arrow indicated right coronary sinus aneurysm. (**B**) Color Doppler flow. Arrow indicated blood flows into the right atrium from the ruptured sinus of Valsalva aneurysm. Ao, aorta; LA, left atrium; PA, pulmonary artery; RA, right atrium, RV, right ventricle, RCSA, right coronary sinus aneurysm.

Initially, the patient presented with sudden chest pain, described as persistent, non-radiating, dull pain, followed by palpitations and headache, accompanied by shortness of breath and cough with a small amount of phlegm, which could not be relieved by rest. He consulted at the local hospital for treatment and was admitted to the intensive care unit (ICU). After admission, he developed anuria and mixed acidosis, which were difficult to correct. His vital signs were as follows: heart rate of 138 beats/min, respiratory rate of 32 breaths/min, blood pressure of 105/38 mmHg (after continuous infusion of high-dose norepinephrine and dobutamine), central venous pressure (CVP) of 9 mmHg, and oxygen saturation of 100% (oxygen absorption flow rate at 4 L/min). Bedside color echocardiography showed an aneurysm of the right coronary sinus of the aorta, which ruptured into the right atrium. The width of the base was 24 mm, the height was 15 mm, and the rupture point was 7 mm. A continuous shunt was observed on color Doppler flow imaging (CDFI) (Figure 1). Left ventricular ejection fraction was 63%. Spectral Doppler showed that the aortic valve jet velocity was normal. CDFI showed that there was no abnormal blood flow across the atrial septum. Minimal aortic valve regurgitation, mild tricuspid regurgitation with a regurgitant area of approximately 3.8 cm^2^ and a maximum velocity of 2.8 m/s, and a pulmonary artery systolic pressure (PASP) of 35 mmHg were observed. The patient was diagnosed with a ruptured sinus of Valsalva aneurysm into the right atrium.

**Figure 2:**
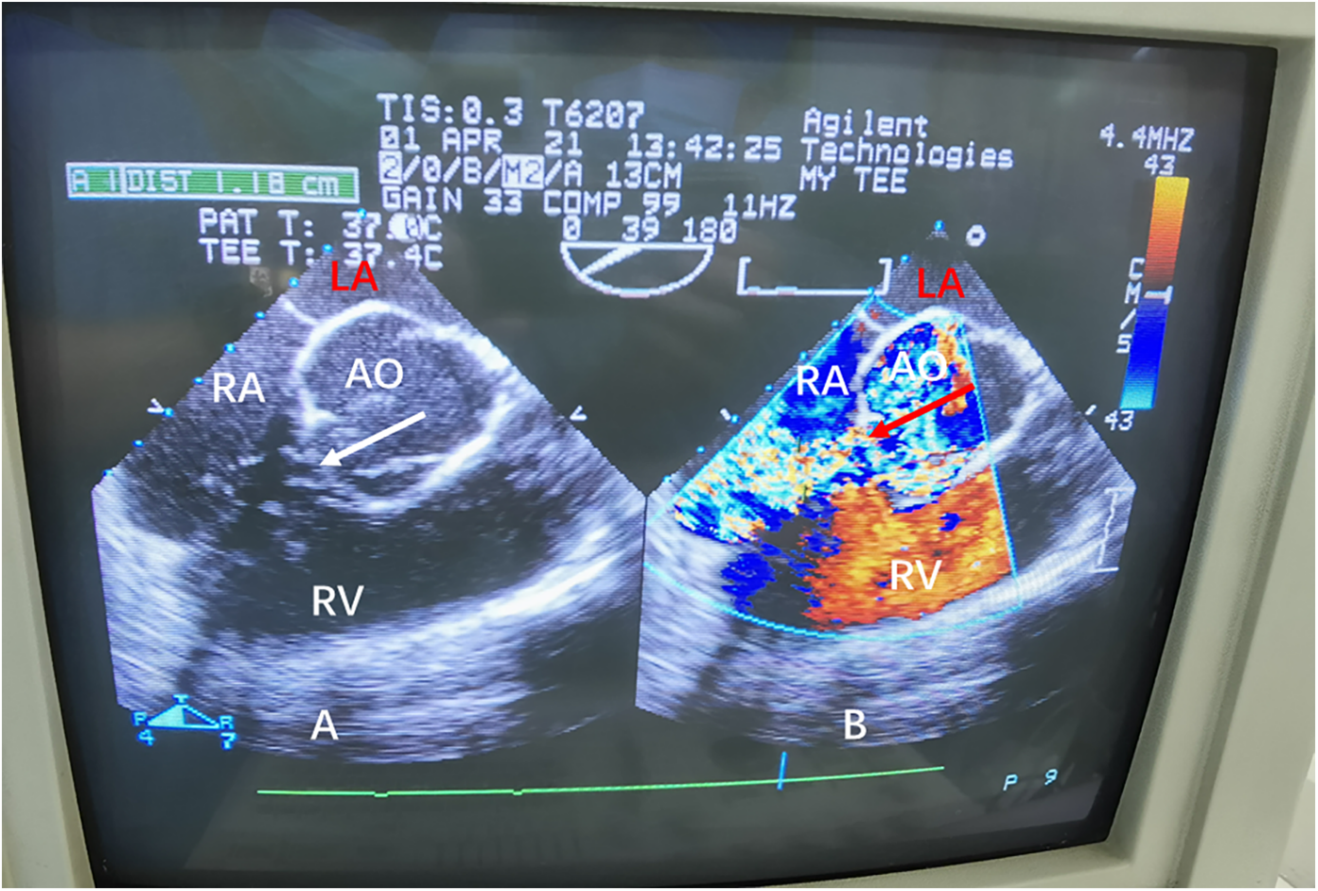
(**A**) Transesophageal echocardiography demonstrating the characteristic “windsock appearance” of the sinus of Valsalva aneurysm. (**B**) Color Doppler flow demonstrating the large left-to-right shunt (arrow) from the ruptured sinus of Valsalva aneurysm into the right atrium. Ao, aorta; LA, left atrium; RA, right atrium; RV, right ventricle.

The local hospital contacted our hospital for consultation. After discussion, the plan was to transfer the patient immediately to our hospital for emergency ruptured sinus of Valsalva aneurysm repair. Considering that the patient's condition was critical and the hemodynamics were still unstable after administration of a large amount of vasoactive drugs, bedside VA-ECMO was performed immediately using 15-French arterial, 8-French arterial backflow, and 21-French venous cannulae via an open right femoral cutdown, achieving flows of 3 L/min. At this time, the patient's vital signs were as follows: heart rate of 130 beats/min, respiratory rate of 22 breaths/min, blood pressure of 109/47 mmHg, and CVP of 29 mmHg. The ECMO flow and dobutamine dose were adjusted to reduce shunting (Table 1). After establishment of ECMO, the patient was transferred by ambulance to our hospital for emergency surgery. Transesophageal echocardiography demonstrated a right sinus of Valsalva aneurysm rupture into the right atrium (Figures 2A,B, Supplementary Video 1).

**Table 1:** Changes in the relationship between mechanical circulation support flow and hemodynamics.

	Pre-ECMO	ECMO flow 1	ECMO flow 2	Post-CPB	Weaning from ECMO
MAP (mmHg)	57	54	58	73	85
CVP (mmHg)	9	29	20	6	12
Mechanical circulation support flow (L/min)	–	3	1.8	3.5	–
Dobutamine (ug/kg/min)	10	6	15	0	5

CVP, central venous pressure; ECMO, extracorporeal membrane oxygenation; MAP, mean arterial pressure.

**Figure 3:**
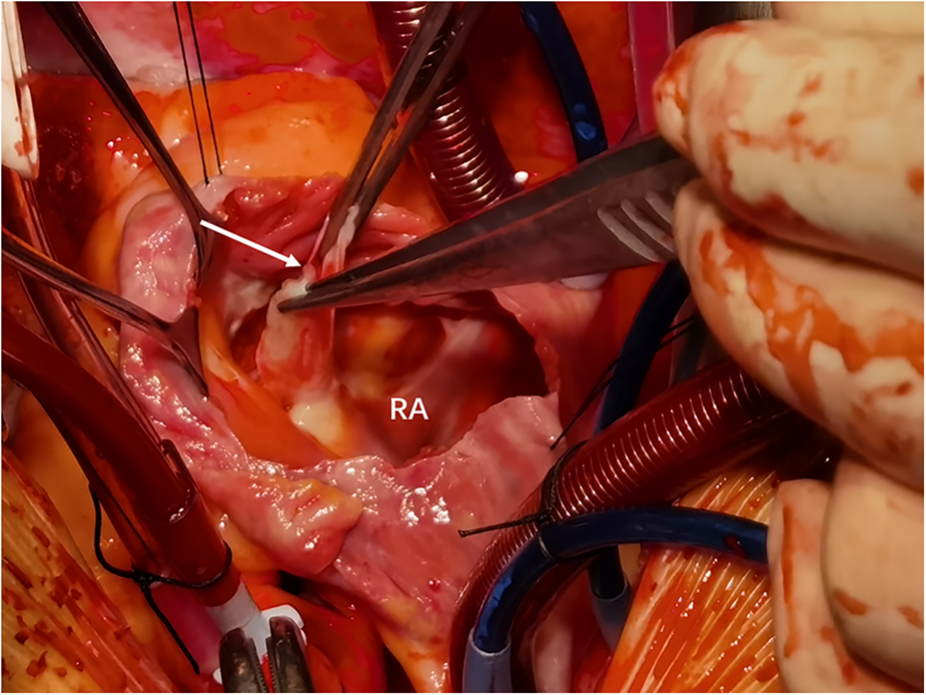
Sinus of Valsalva aneurysm (arrow) ruptured into the right atrium. RA, right atrium.

**Figure 4:**
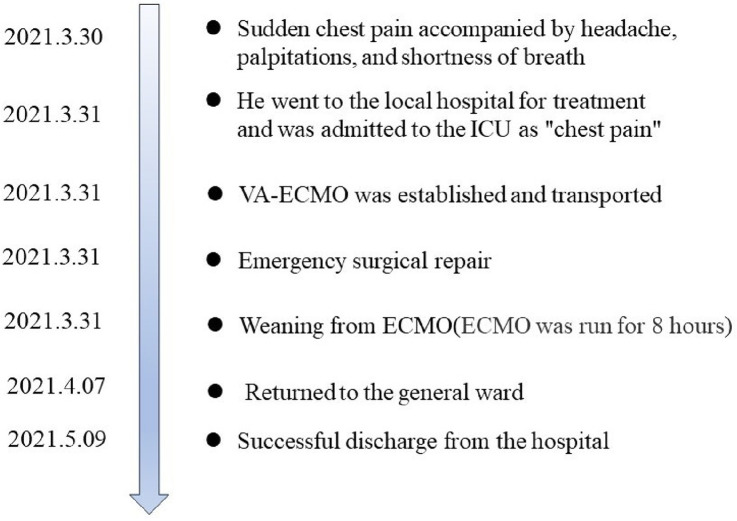
Timeline for this case. ICU, intensive care unit; VA-ECMO, veno-arterial extracorporeal membrane oxygenation.

The patient underwent emergency surgical repair. A median sternotomy was performed, and after systemic heparinization, cardiopulmonary bypass was established with bicaval cannulation. A cardioplegia cannula was inserted into the aortic root, and a left ventricular vent was inserted into the right superior pulmonary vein. The patient was then placed under moderate hypothermia. After an aortic cross-clamping, antegrade and retrograde cross perfusion of histidine-tryptophan-ketoglutarate (HTK) cardioplegia solution were performed. The right atrium was incised, and the aortic valve was exposed through an oblique incision at the aortic root. Exploration showed that the aortic sinus was grossly dilated and penetrated into the right atrium, with a rupture size of approximately 1.2 × 1 cm (Figure 3). The rupture was repaired with a pericardial patch sutured intermittently using 4-0 polypropylene. No leak was detected after the repair. The aortic root and the right atrial incision were then closed using 5-0 polypropylene sutures. Cardiac circulation was then resumed, cardiopulmonary bypass was discontinued when the blood pressure stabilized. Ventilator parameters were as follows: tidal volume of 500 ml, respiratory rate of 14 breaths/min, positive end-expiratory pressure of 5 cm H_2_O, inspired oxygen concentration of 60%. The following vital signs were observed: blood pressure of 125/65 mmHg, heart rate of 80 beats/min, pulse oxygen saturation of 100%. Blood gas analysis showed the following results: pH of 7.45, PCO_2_ of 39 mmHg, PO_2_ of 270 mmHg, glucose of 8 mmol/L, lactic acid of 1.10 mmol/L, HCO_3_ of 27.1 mmol/L, and hemoglobin (Hgb) of 94 g/L. Urine output was 3 ml/kg body weight/h. The patient's hemodynamic status stabilized, ECMO was discontinued. Then protamine was administered. The patient was hooked to ECMO for 8 h. He returned to the surgical ICU after surgery and to the general ward after 7 days. He was successfully discharged after 40 days (Figure 4). After 2 years of follow-up, the patient's heart structure and function were normal. He had good exercise tolerance and no evidence of heart failure.

## Comment

Ruptured congenital sinus of Valsalva aneurysm is a rare disease that can cause heart palpitations, chest pain, breathing difficulties, and even sudden death. It often ruptures into the right atrium or right ventricle, causing blood from the aorta to pass through the sinus and back to the right atrium or right ventricle, thereby increasing the right heart load and causing acute right heart failure. According to relevant reports, once a ruptured sinus of Valsalva aneurysm is diagnosed, the average survival time without surgical treatment is 1–2 years (1). Therefore, patients with ruptured sinus of Valsalva aneurysms should be actively treated with surgery, and most patients who undergo surgical repair can survive for a long time (5). In this case, the sinus of Valsalva aneurysm ruptured into the right atrium, resulting in cardiogenic shock and requiring emergency surgical treatment. However, the local hospital where the patient was initially admitted lacked the capability to perform this operation and he needed to be transferred to a facility with access to advanced medical care. The patient in this case was in critical condition, and the risks associated with remote referral were high. As a means of circulatory support, ECMO can ensure tissue perfusion and prevent tissue and organ hypoxia that may be caused by possible cardiac arrest during transport (6). In this case, after the sinus of Valsalva aneurysm ruptured into the right atrium, the patient's diastolic blood pressure decreased significantly. Chest tightness and pain increased in severity, indicating that the cardiac blood supply was relatively insufficient and the heart function was impaired, which may increase the risk of cardiac arrest during transport. Therefore, we performed VA-ECMO prior to remote transport in this case, since the main concern was the difficulty of establishing ECMO if cardiac arrest occurred during transport. Due to the presence of a shunt, it would be difficult to ensure the quality of cardiopulmonary resuscitation, with potentially fatal consequences for the patient. In this case, VA-ECMO was used as a safety measure to ensure tissue and organ perfusion if the patient suffered cardiac arrest. Based on the proven ECMO technology at our center, the benefits of this approach outweigh the potential complications. Therefore, ECMO is an effective means to ensure the safety of this patient.

ECMO assistance can prevent the occurrence of tissue hypoperfusion in patients with severe cardiogenic shock or cardiac arrest during transport. However, blood flow in peripheral ECMO established through the femoral arteriovenous system is retrograde; this increases cardiac afterload, thus increasing the flow of aortic blood to the right atrium through the ruptured sinus (7). After establishment of VA-ECMO, the patient's CVP increased from 9 to 29 mmHg, and the right atrial pressure increased significantly. Therefore, in this case, methods to reduce ECMO flow and strengthen the heart were adopted to minimize the adverse effects of ECMO. Singh et al. reported a successful case of emergency surgical treatment after ECMO for a sinus of Valsalva aneurysm with rupture to the right atrium leading to cardiac arrest (8). Rapid establishment of cardiopulmonary bypass and repair of ruptured sinus of Valsalva aneurysm are key.

## Conclusions

ECMO transport is an effective way to ensure the safety of patients with severe shock due to a ruptured sinus of Valsalva aneurysm to the right heart. The increase in right cardiac load should be countered by adjusting the ECMO flow, combined with use of cardiotonic drugs. Rapid establishment of cardiopulmonary bypass and repair of ruptured sinus of Valsalva aneurysms are key to successful treatment. Further research is needed to investigate the optimal ECMO flow and decompression methods in the future.

## Data Availability

The original contributions presented in the study are included in the article/**Supplementary Materials**, further inquiries can be directed to the corresponding authors.
